# IgV somatic mutation of human anti–SARS-CoV-2 monoclonal antibodies governs neutralization and breadth of reactivity

**DOI:** 10.1172/jci.insight.147386

**Published:** 2021-05-10

**Authors:** Mayara Garcia de Mattos Barbosa, Hui Liu, Daniel Huynh, Greg Shelley, Evan T. Keller, Brian T. Emmer, Emily Sherman, David Ginsburg, Andrew A. Kennedy, Andrew W. Tai, Christiane Wobus, Carmen Mirabeli, Thomas M. Lanigan, Milagros Samaniego, Wenzhao Meng, Aaron M. Rosenfeld, Eline T. Luning Prak, Jeffrey L. Platt, Marilia Cascalho

**Affiliations:** 1 Department of Surgery,; 2 Department of Urology,; 3 Biointerfaces Institute,; 4 Department of Internal Medicine,; 5 Life Sciences Institute,; 6 Departments of Human Genetics and Pediatrics and Howard Hughes Medical Institute,; 7 Department of Microbiology and Immunology, and; 8 Vector Core, Biomedical Research Core Facilities, University of Michigan, Ann Arbor, Michigan, USA.; 9 Department of Medicine, Henry Ford Health Systems, Detroit, Michigan, USA.; 10 Department of Pathology and Laboratory Medicine, Perelman School of Medicine, University of Pennsylvania, Philadelphia, Pennsylvania, USA.

**Keywords:** COVID-19, Immunology, Immunoglobulins, Molecular biology

## Abstract

Abs that neutralize SARS-CoV-2 are thought to provide the most immediate and effective treatment for those severely afflicted by this virus. Because coronavirus potentially diversifies by mutation, broadly neutralizing Abs are especially sought. Here, we report a possibly novel approach to rapid generation of potent broadly neutralizing human anti–SARS-CoV-2 Abs. We isolated SARS-CoV-2 spike protein–specific memory B cells by panning from the blood of convalescent subjects after infection with SARS-CoV-2 and sequenced and expressed Ig genes from individual B cells as human mAbs. All of 43 human mAbs generated in this way neutralized SARS-CoV-2. Eighteen of the forty-three human mAbs exhibited half-maximal inhibitory concentrations (IC_50_) of 6.7 × 10^–12^ M to 6.7 × 10^–15^ M for spike-pseudotyped virus. Seven of the human mAbs also neutralized (with IC_50_ < 6.7 × 10^–12^ M) viruses pseudotyped with mutant spike proteins (including receptor-binding domain mutants and the S1 C-terminal D614G mutant). Neutralization of the Wuhan Hu-1 founder strain and of some variants decreased when coding sequences were reverted to germline, suggesting that potency of neutralization was acquired by somatic hypermutation and selection of B cells. These results indicate that infection with SARS-CoV-2 evokes high-affinity B cell responses, some products of which are broadly neutralizing and others highly strain specific. We also identify variants that would potentially resist immunity evoked by infection with the Wuhan Hu-1 founder strain or by vaccines developed with products of that strain, suggesting evolutionary courses that SARS-CoV-2 could take.

## Graphical abstract

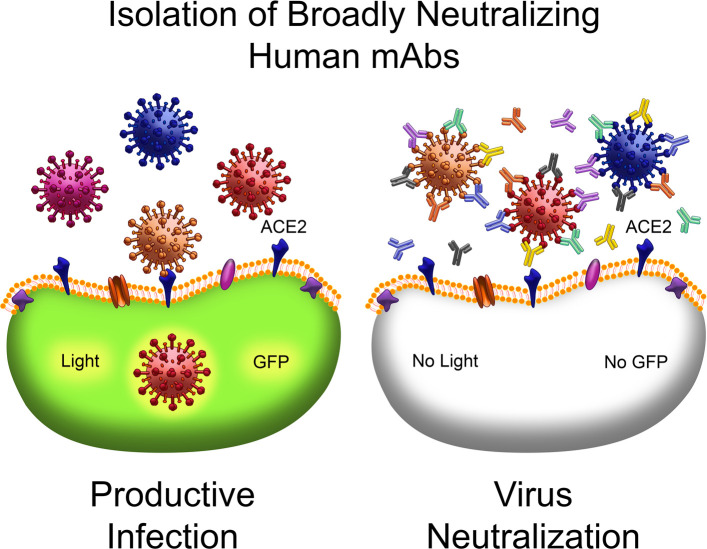

## Introduction

The immune response to SARS-CoV-2 is thought to promote clearance of the virus, recovery from clinical manifestations and protection against reinfection (1, 2). Among products of the immune response to SARS-CoV-2, Abs capable of neutralizing SARS-CoV-2 are believed to be especially important for controlling severe manifestations of the disease (3). Administration of convalescent serum and mAbs against the SARS-CoV-2 spike protein is reported to facilitate and hasten recovery from SARS-CoV-2 pneumonia (NCT04426695, NCT04425629, NCT04592549, and others) (4, 5). It is as-yet unclear whether any of the individual Abs or collections of Abs thus far obtained effectively block variants of SARS-CoV-2 that have already emerged. It is also not clear which among various approaches used to obtain anti–SARS-CoV-2 Abs could rapidly adapt to control novel variants of this virus likely to emerge over time.

Immune serum and isolated Igs have been effective in multiple settings (6–9). However, availability from any given subject or pool of subjects is limited and not standardized. Furthermore, immune sera/plasma contain mixtures of Abs, some of which might enhance the virus, even though there is no evidence so far of enhancement in convalescent serum trials (10). These limitations motivate the development of mAbs, especially human mAbs. Human mAbs are especially desired for at least three reasons. First, human mAbs can be generated directly from immune B cells without the time-consuming engineering needed to replace mouse sequences with human sequences. Second, human mAbs are less immunogenic than mouse mAbs, even after “humanization,” and hence less apt to generate immune complexes, extending their half-life. Third, human mAbs cloned from immune human B cells potentially reflect in vivo selection of cells expressing Abs with antiviral properties (11).

One challenge in developing human mAbs, however, is that the B cells most readily available are peripheral blood B cells and only a small fraction (usually less than 1%) of these are specific for any given antigen (12, 13). Accordingly, production of human mAbs generally requires the screening of numerous B cells, the preponderance producing Abs that do not bind or only weakly bind the antigen of interest. We devised an approach that rapidly bypasses this problem and herein report that isolating human mAbs from memory B cells that bind viral antigen under physiologic conditions yields a high percentage of neutralizing human mAbs.

Another hurdle to developing human anti–SARS-CoV-2 mAbs capable of controlling pathogenicity of the virus is genetic diversification and evolution of the virus. A number of SARS-CoV-2 variants have developed and spread in populations (14). Human Abs directed against mutable viruses, such as HIV, vary greatly in the diversity of strains recognized, and effective control is associated with presence of broadly neutralizing Abs in blood (15, 16). We tested the human mAbs we developed for neutralization of known SARS-CoV-2 strains and for the ability to recognize SARS-CoV-2 spike protein variants that have developed across the world.

We describe here the approach we devised for rapid development of human mAbs and show that this approach can efficiently generate human neutralizing Abs against prevailing strains of SARS-CoV-2. We also show that the approach efficiently generates broadly neutralizing Abs and that the characteristic of broad neutralization is associated with extensive mutation of Ig variable region sequences and likely with selection. As SARS-CoV-2 continues to evolve, effective control of severe disease might best be achieved by employing the strategies we describe to generate mixtures of highly specific and broadly reactive neutralizing Abs.

## Results

### Plasma from convalescent patients neutralize pseudotyped viruses expressing reference and mutant strain spikes.

To develop human mAbs potentially capable of neutralizing multiple strains of SARS-CoV-2, we obtained B cells from subjects who had experienced symptomatic infection with SARS-CoV-2 at least 4 weeks before and who had successfully cleared active virus. Although T cells and anti–SARS-CoV-2 Ab likely contributed to viral clearance, the interval of 4–8 weeks potentially allows somatic hypermutation (SHM) and selection to optimize the affinity of anti–SARS-CoV-2 Ab, increase the frequency of memory B cells circulating in blood and potentially minimizes the frequency of B cells producing enhancing Ab (10). Based on these criteria, we identified 12 convalescent volunteers (pertinent characteristics of the subjects are listed in the Methods section) as sources of the B cells for generation of mAbs.

To verify that the subjects had mounted protective Ab responses against SARS-CoV-2 and to compare mAbs generated using B cells from those subjects, we devised a sensitive and stable assay for SARS-CoV-2 neutralization (Supplemental Figure 1; supplemental material available online with this article; https://doi.org/10.1172/jci.insight.147386DS1). We modeled SARS-CoV-2 by introducing a SARS-CoV-2 spike protein on the surface of a third-generation HIV lentivirus backbone to produce spike-pseudotyped lentiviruses. To this end, a 19 AA C-terminal deletion of the S gene, encoding the spike protein from the Wuhan Hu-1 or mutant SARS-CoV-2 strains, was transduced in human embryonic kidney 293T/17 (293T/17) cells. The pseudotyped virus produced by the cells was used to assay neutralization. Spike-pseudotyped lentiviruses infected 293T cells expressing angiotensin-converting enzyme 2 (ACE2), the SARS-CoV-2 receptor, in an ACE2-dependent manner and as efficiently as control VSV-G–pseudotyped viruses (Supplemental Figures 1 and 2; see complete unedited blots in the supplemental material). In this assay, 4 of the 12 convalescent plasmas had 50% neutralization titers (ID_50_) above 1:100, indicating that a fraction of the subjects had appreciable neutralizing responses to SARS-CoV-2 infection.

### Isolation of memory B cells with high avidity for SARS-CoV-2 spike protein.

Human mAbs against SARS-CoV-2 have been generated by single-cell cloning of Ig genes from peripheral blood B cells (3, 4, 17–21). One surprising finding was that anti–SARS-CoV-2–neutralizing Abs were not heavily mutated. We reasoned that we could optimize the development of biologically active human Abs with high neutralizing potency by using as the source of the Ig DNA memory B cells with antigen receptors that avidly bind antigen of interest under physiologic conditions. Accordingly, peripheral blood mononuclear cells were incubated for 72 hours in medium containing CpG, CD40L, IL-2, IL-15, and IL-21, which favors survival and expansion of memory B cells (22). The cultured cells were then transferred to wells containing SARS-CoV-2 S1 antigen in native configuration and incubated for 24 hours, followed by isolation of antigen-specific B cells by panning at 37°C (11, 20, 23). Panned cells were then cultured with the cytokine cocktail for an additional 48 hours. The specificity of the B cells was confirmed by ELISPOT (Supplemental Figure 3).

In samples from 6 recovered subjects, the frequency of IgG-producing spike-specific B cells as a fraction of the total IgG-producing cells increased with the neutralization titer of the plasma. Thus, following 6 days of culture, 8%, 2%, and 13.3% (of subjects 2, 3, and 4, respectively) of IgG^+^ memory B cells secreted Abs that bound to the spike protein. In contrast, healthy controls or previously infected patients with low plasma neutralization titer had few or no spike-specific B cells.

### Spike-specific B cells encode mutated Abs.

**Figure 1:**
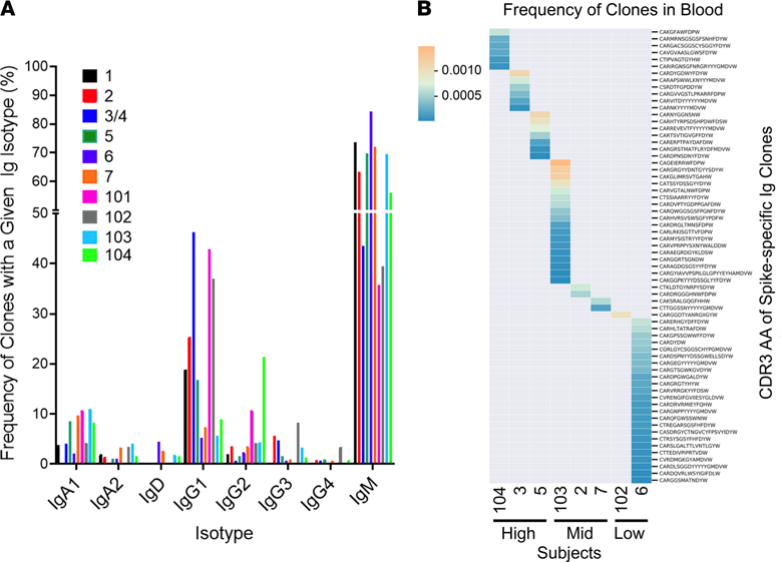
Properties of spike-specific Ig sequences. Immune repertoire profiling data were generated by single-cell sequencing using the 10× platform on B cells that were panned for binding to the SARS-CoV-2 spike protein (as detailed in Methods). Additional data on Ab heavy chain gene rearrangements were generated on bulk PBMC gDNA (see Methods). (**A**) Frequency of spike-specific single-cell sequenced clones separated by Ig isotype. Each color indicates a subject, and the height of each bar represents the frequency of spike-specific clones expressing the given isotype in that subject. (**B**) String plot of spike-binding clones that were also found in the bulk blood libraries. Each row represents a clone, each column represents a subject, and the intensity of each cell shows the copy number fraction of the associated spike-specific clone in the blood.

We performed single-cell sequencing of spike-specific B cells obtained from 11 subjects followed by paired-end single index Ig sequencing. Clonally related B cells were defined as having the same IGHV gene, IGHJ gene, and CDR3 length and 85% identity in the CDR3 amino acid sequence, as described previously (24, 25). We obtained more than 6000 spike-specific Ig clones. The frequency of IgM clones varied from a minimum of 35.7% to a maximum of 84.3%, the frequency of IgG isotypes varied between 8.0% and 53.7%, and the frequency of IgA isotypes varied between 1.4% and 15.1% (Figure 1A). Average IgA usage was almost 2-fold higher in subjects with higher plasma neutralization titers (ID_50_ > 1:50; 9.1%) compared with subjects with plasma neutralization titers lower than 1:50 (5.5%) (Figure 1A). In contrast, the frequencies of IgM- and IgG1-encoding isotypes did not differ significantly between individuals with higher neutralization titers and those with lower neutralization titers.

**Figure 2:**
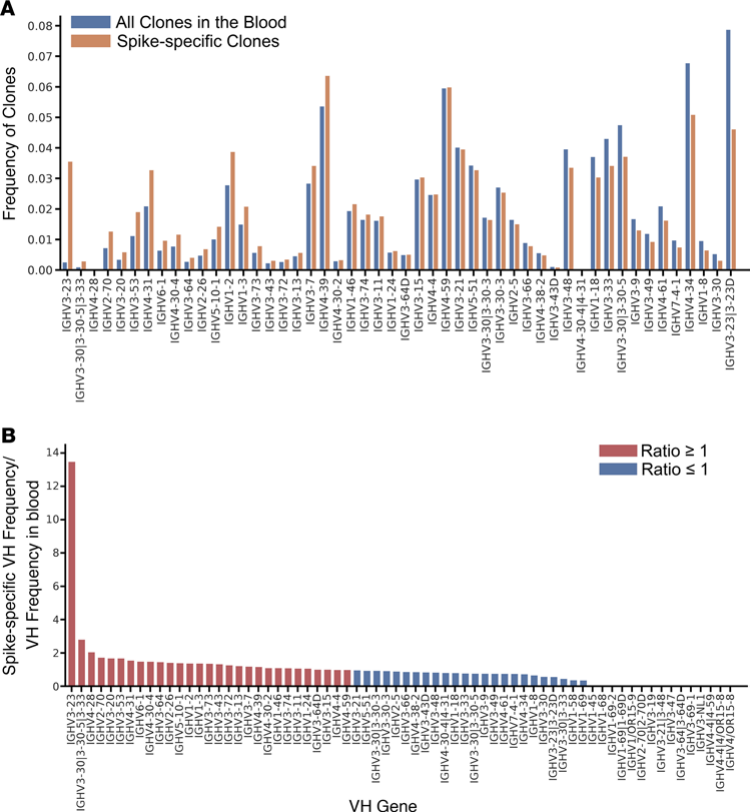
Comparison of the spike-specific VH repertoire and the VH repertoire in the blood. IgH sequences were obtained from nonpanned blood B cells by NGS and from spike-panned B cells, as explained in the legend of Figure 1. (**A**) Number of clones using the top 50 most frequent VH genes in spike-specific clones and in all clones found in the blood. Each column indicates a VH gene, blue bars indicate the frequency of the given VH (by clone) in the blood, and the oranges bar indicate the frequency among spike-specific clones. (**B**) Fold change of VH gene frequencies in spike-enriched Ig clones as compared with VH gene frequencies in the blood. The height of each bar indicates the ratio of VH gene usage of spike-specific clones to that of other clones in the blood with the same VH. Red indicates a higher frequency in spike-binding clones and blue indicates lower frequency in spike-binding blood clones

**Figure 3:**
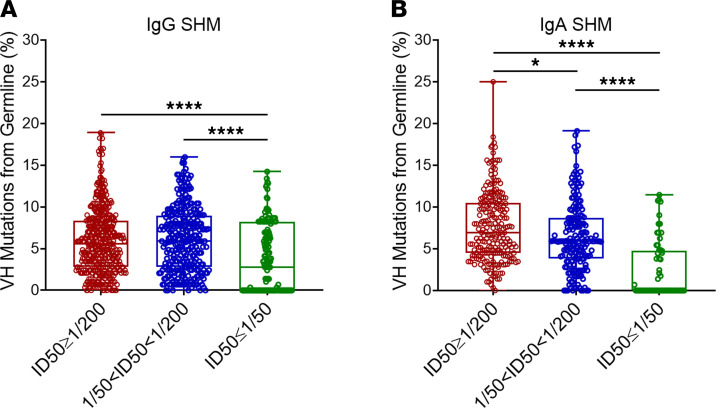
Somatic mutation of spike-specific VH genes according to the subjects’ level of virus neutralizing Abs in plasma. The frequency of mutated IgG (**A**) or IgA (**B**) VH sequences in relation to germline in subjects grouped by the level of virus neutralizing Abs. ID_50_ ≤1:50, low neutralizing; 1:50 <ID_50_ < 1:200, mid neutralizing; ID_50_ >1:200, high neutralizing. Analysis was by 2-tailed Mann Whitney test function of Prism 9. Data represent mutations in the variable exons of all single-cell IgG or IgA spike-panned B cell clones. Mean ± SEM.

To determine the frequency of spike-binding Ig clones in the blood, paired-end bulk sequencing was performed on the same donors, as described in Methods. Spike-specific Ig clones were relatively rare in the blood (less than 0.1% of all B cell clones) (Figure 1B). Some VH3 family members appeared to be enriched (more than 2-fold) in the spike-specific libraries (IGHV 3-30, IGHV 3-13, IGHV 3-23, IGHV 3-72, IGHV 3-73, and IGHV 3-66) (Figure 2). Other studies (26–28) found that 10% of SARS-CoV-2–neutralizing Abs were encoded by IGHV 3-53, at a frequency at least 4 times higher than that observed in the blood of naive healthy individuals (29, 30). These Abs had few somatic mutations, and Yuan et al. (26) found that recognition of ACE2 by the RBD was mediated by germline-encoded residues (NY motif at VH residues 32 and 33 in the CDRH1 and an SGGS motif at VH residues 53 to 56 in CDRH2), suggesting that neutralization potency was dissociated from affinity maturation. In contrast, our single-cell data suggest that strongly neutralizing Abs are somatically mutated (Figure 3). In particular, the frequency of IgG or IgA clones that harbored VH sequences with SHM increased with serum neutralization titers. For sequences with greater than 10% SHM, contingency analysis between weakly and strongly neutralizing groups by χ^2^ test yielded *P* = 0.0066 for IgG and *P* = 0.0022 for IgA. We also found that only 1 of 274 IgA clones obtained from subjects with high neutralizing serum (ID_50_ > 1:200) was unmutated, while 44 of 214 (20.6%) IgA clones obtained from subjects with low neutralizing serum (ID_50_ < 1:50) were germline and had, on average, 2 AA smaller CDR3 regions. These findings suggested that adaptive IgA/IgG responses contribute to neutralization.

### Generation of SARS-CoV-2 highly neutralizing Abs.

**Figure 4:**
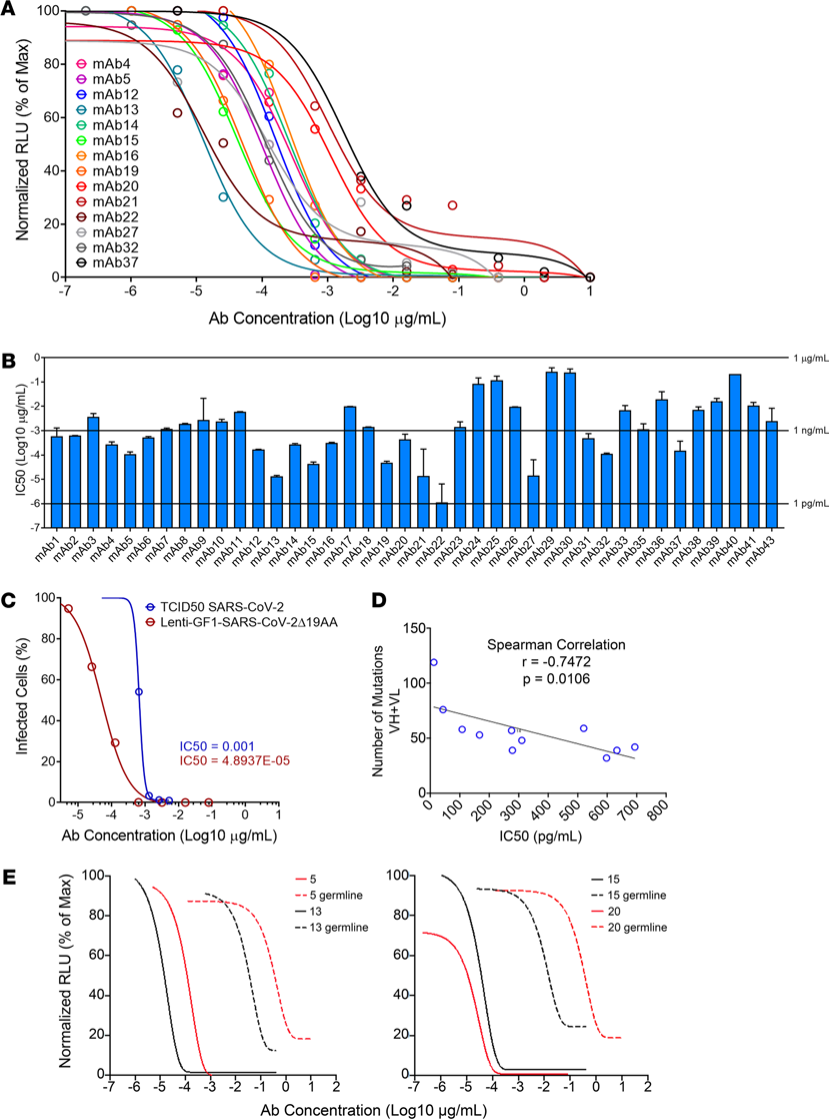
Neutralization of virus pseudotyped with SARS-CoV-2 Wuhan Hu-1 spikes by human mAbs. Highly mutated mAbs were cloned and expressed on 293T/17 cells. The supernatants were collected, and the amount of IgG was measured by ELISA. Serially diluted Abs were incubated with viruses pseudotyped with the reference (Wuhan Hu-1) or mutant spikes, and infection of 293T-ACE2 cells was assessed by luminescence. The Ab concentration that inhibits 50% of infection (IC_50_) was calculated for each sample. (**A**) The neutralization curves are depicted for 40 of 43 mAbs isolated to illustrate the breath of neutralization potency. Curves were obtained from 10 serial dilutions measured in duplicate. (**B**) The IC_50_ for each of the isolated mAbs. Error bars represent mean ± SEM. (**C**) The neutralization curve of mAb 19 with SARS-CoV-2 live virus, Washington USA-WA1/2020 clinical isolate, and SARS-CoV-2 Wuhan Hu-1 spike-pseudotyped virus. Results were obtained from 8 serial dilutions. (**D**) The linear correlation of IC_50_ and number of mutations in the VH and VL genes for the 11 most potent mAbs. Analysis was by the simple linear regression followed by Spearman’s r test functions of Prism 9. (**E**) Neutralization curves of viruses pseudotyped with SARS-CoV-2 Wuhan Hu-1 spike of mAbs 5, 13, 15, and 20 mutated and in germline configuration. Neutralization data curves were analyzed by the nonlinear fit function of Prism 9 and the absolute IC_50_ was calculated when possible. Data reflect typical plots from at least 2 independent experiments.

To generate human mAbs specific for SARS-CoV-2 spike, we cloned the IgH and IgL variable regions 3′ to the human IgG1 constant region (27, 28). We selected IgV sequences of IgG or IgA Abs, with mutated VH regions (>5% compared with their closest germline), that were represented in the sequencing pool by multiple copies. We expressed Ig VH and VL pairs generating 43 recombinant spike-specific Abs from SARS-CoV-2–specific B cells (27, 28). Ab concentrations were determined by ELISA, and neutralization potency was determined against pseudotyped viruses, as indicated on Supplemental Figure 1. All 43 mAbs synthesized neutralized Wuhan Hu-1 spike-pseudotyped virions; 18 of 43 Abs neutralized spike-pseudotyped viruses with extremely high potency, with half-maximal inhibitory concentration (IC_50_) of less than 1 ng/mL (6.7 × 10^–12^ M). Four of the mAbs (mAbs 13, 21, 22, and 27) had IC_50_s close to 1 pg/mL (6.7 × 10^–15^ M) (Figure 4, A and B). These IC_50_s are comparable to the most powerful Abs produced by Regeneron (4) and by others (3, 21, 23, 29) and tested by a pseudotyped virus assay similar to ours. mAb 19 did also neutralize live WA1 virus with comparable IC_50_s (Figure 4C), indicating, as others have shown (17), that IC_50_s determined by pseudotyped virus assay closely reflect neutralization potencies against live viruses. Neutralization potency increased with the number of mutations in the VH and VL exons of the 11 mAbs with the lowest IC_50_s (Figure 4D), suggesting that affinity maturation contributes to high neutralization potency. To directly test the contribution of SHM to neutralization potency, we reverted VH and VL sequences of mAbs 5, 13, 15, and 20 to their germline configuration and tested their neutralization potency. Figure 4E shows that germline-encoded Abs neutralized pseudotyped viruses expressing the Wuhan Hu-1 spike much less efficiently, by at least 1000-fold, when compared with their mutated counterparts, indicating that SHM contributed to increased neutralization efficiency in these Abs.

### SARS-CoV-2 highly neutralizing Abs are broadly reactive.

To determine if natural infection induced broadly neutralizing Abs, we tested neutralization of pseudotyped viruses with spike protein variants in the S1 N-terminal domain (H49Y, V247R, V367F, R408I), in the receptor-binding domain (V483A, H519Q, A520S), and in the S1-C-terminal domain (D614G). Of these variants, A520S and D614G have notably greater infectivity than WT Wuhan Hu-1 (30). Testing of plasma revealed notable variation in specificity for variants. While plasma 3 exhibited broad neutralization, plasma from subject 102 did not neutralize at all viruses pseudotyped with the Wuhan-1, D614G, S247R, or H49Y spikes but neutralized moderately H519Q, and A520S spike-pseudotyped viruses, suggesting that the plasma contains Abs with narrow specificity for the mutant RBD (Supplemental Figure 4, A and B). In another notable result plasma from subject 4 neutralized H519Q spike-pseudotyped viruses 100-fold more potently than any other spike-pseudotyped viruses (Supplemental Figure 4, A and B). However, serology alone could not determine whether these differences reflected differences in the breadth of neutralization of individual Abs or merely the diversity of Abs present in the plasma.

**Figure 5:**
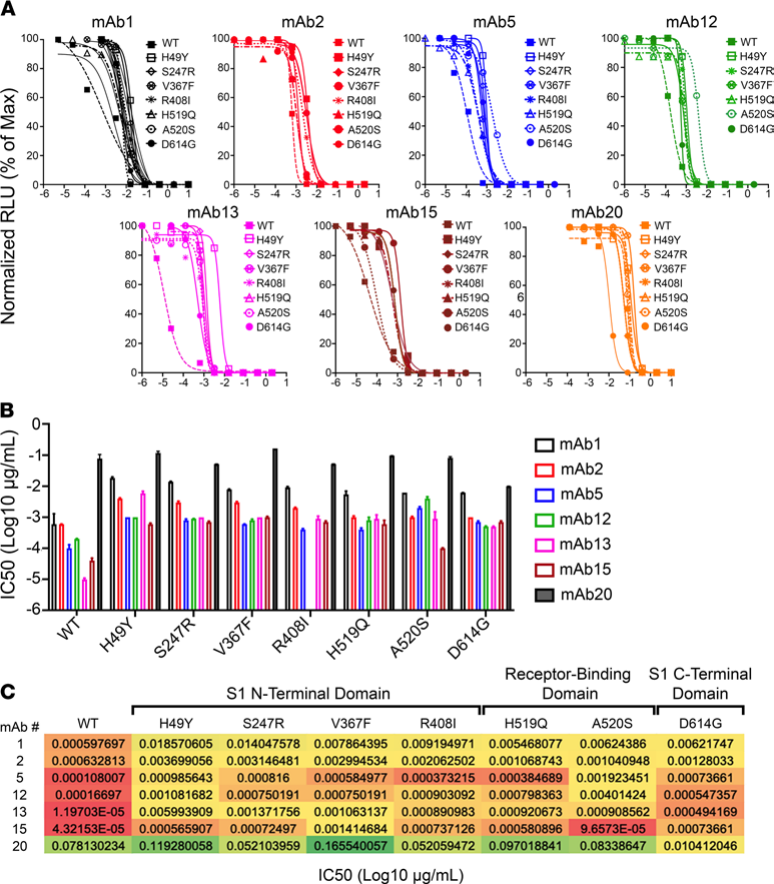
Neutralization of virus pseudotyped with SARS-CoV-2 mutant spikes by spike-specific highly mutated human mAbs. (**A**) Neutralization curves of viruses pseudotyped with SARS-CoV-2 variant spikes for mAbs 1, 2, 5, 12, 13, 15, and 20. (**B**) mAb 50% inhibitory concentration (IC_50_) for SARS-CoV-2 variant spikes. Mean ± SEM. (**C**) The monoclonal IgG1 Ab IC_50_ for SARS-CoV-2 spikes encoding the following mutations: H49Y, S247R, V367F, R408I, H519Q, A520S, and D614G. Data were analyzed by the nonlinear fit function of Prism 9, and the absolute IC_50_ was calculated when possible. Data reflect a typical plot from 3 independent experiments. Colors reflect IC_50_: lowest values are shown in red in a gradient to the highest values, shown in green.

**Figure 6:**
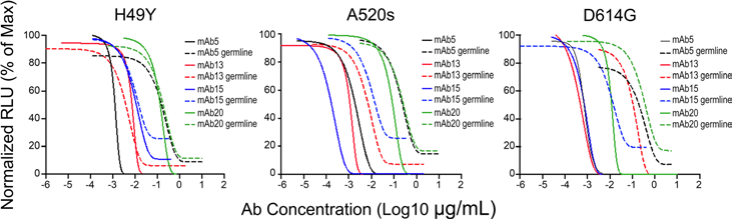
Neutralization of virus pseudotyped with SARS-CoV-2 mutant spikes by mature mAb and in germline configuration. The neutralization curves of viruses pseudotyped with SARS-CoV-2 H49Y, A520S and D614G spikes of mAbs 5, 13, 15, and 20 mutated and in germline configuration are shown. Data were analyzed by the nonlinear fit function of Prism 9, and the absolute IC_50_s calculated when possible. Data reflect a typical plot from 3 independent experiments.

To distinguish between these two possibilities, we next tested the mAbs with the highest potency (IC_50_ < 6.7 × 10^–12^ M; mAbs 1, 2, 5, 13, 15, and 20) for breadth of neutralization. Most mAbs with high potency against Wuhan Hu-1 spike-pseudotyped virus also neutralized variants of that strain but with 10- to 100-fold less potency (Figure 5A). The most powerful mAbs were the least cross-reactive. For example, mAb 13 neutralized virus with Wuhan Hu-1 spike at an IC_50_ of 8.3 × 10^–14^ M but exhibited approximately 100-fold less neutralizing potency against the mutants tested (Figure 5). As another example, mAb 15 neutralized Wuhan Hu-1 at an IC_50_ of 3.0 × 10^–13^ M but had 10- to 100-fold lower potency against most mutants. mAbs 1, 2, and 5, were notably potent against Wuhan Hu-1 (IC_50_ varying from 7.4 × 10^–13^ for mAb 5 to 4.1 × 10^–12^ M and 4.3 × 10^–12^ M, for mAbs 1 and 2, respectively) and also had 10- to 100-fold lower potency against most mutants. However, mAb 20 neutralized Wuhan Hu-1 at IC_50_ of 5.3 × 10^–10^ M but neutralized D614G mutants with higher potency at IC_50_ of 3.5 × 10^–10^ M (Figure 5). Neutralization of D614G mutants by mAb 20 (and also by mAbs 5, 13, and 15) depended on somatic mutations, as the germline-encoded Ab was at least 100-fold less efficacious (Figure 6). Interestingly, germline mAbs 13, 15, and 20 neutralized H49Y spike mutants as well their mutated counterparts (Figure 6), indicating that the H49Y mutation does not perturb the binding epitope responsible for neutralization. Neutralization of A520S spike mutants by mAbs 5, 13, and 15 but not by mAb 20 depended on somatic mutations (Figure 6). Data indicate that SHM of Abs is necessary for high-potency neutralization but that some broad-reactivity may be explained by properties afforded by the germline precursors of the mature mutated Abs.

## Discussion

We adapted an approach, originally developed to isolate donor-specific B cells from the blood of transplant recipients, to the rapid and efficient generation of human mAbs with high- and broadly neutralizing potency against SARS-CoV-2. Our approach includes a brief incubation of PBMCs with a cytokine cocktail and exposure to antigen in native form in cell culture to selectively recover memory B cells specific for the antigen of interest (22). Using avidity binding to immobilized native antigen as an initial step, we avoided time-consuming screening of unselected B cells and were able to focus rapidly on selecting B cells with optimized specificities. In this way, we generated highly neutralizing mAbs without any further screening, in less than 6 weeks. All 43 Abs that we produced had high neutralization capability against SARS-CoV-2 viruses. Although the Abs generated in this way have not been tested for clinical efficacy, the potency of neutralization exceeds by orders of magnitude the potency of neutralization of human mAbs currently in clinical trials (NCT04426695, NCT04425629, NCT04592549, and others) (4, 5).

Even more important may be the ability of these Abs to neutralize mutant strains. It is clear that diversification of SARS-CoV-2 has already occurred in populations. The CoV-GLUE database (14) reports more than 5300 distinct nonsynonymous mutations in the gene encoding the spike in natural SARS-CoV-2 populations. The significance of many variants for infectivity, spreading, and immune evasion is at present unknown, but recent research suggests that some mutations may increase severity of disease and/or virus infectivity. Becerra-Flores et al. (31) suggested a causal link between D614G variants and increased fatality in patients with COVID-19. Qianqian Li et al. (30) found that the D614G and the RBD A520S spike variant increased infectivity of pseudotyped viruses. Recently, Hou et al. (32) found that the D614G mutation increased virus infectivity, competitive fitness, and transmission in primary human cells and in animal models.

Although we do not yet know if immunity drives virus evolution evidence supports this possibility. Variants that are resistant to neutralizing Abs have increased in frequency since the beginning of the pandemic (33, 34). Baum et al. (4) and Weisblum et al. (33) report that neutralizing Abs readily select for resistant virus after in vitro passaging, even though coronavirus immune escape is not yet known to influence pathogenicity in individual patients. In theory, broadly neutralizing Abs are less forgiving of diversification and hence variants in a population could be less likely to emerge when broadly neutralizing Abs are present. Although not the main objective of our work, and even if variants may arise owing to increasing virus fitness, the results presented here offer a glimpse at viral variants that might evade typical Ab responses and hence increase in frequency as SARS-CoV-2 evolves. Our results indicate that D614G and A520S variants are more resistant to neutralizing Abs than viruses with the Wuhan Hu-1 spike, suggesting that immunity elicited against the Wuhan Hu-1 (the prevalent strain in Michigan at the time of sample collection) might fuel escape variants. It is noteworthy that some of the described public Abs against SARS-CoV-2 have minimal levels of SHM (35). This finding, similar to what has been described for other newly encountered pathogens (36), suggests that the naive repertoire contains Abs that can bind and neutralize the virus. Consistent with this, Abs (such as mAb 20) have modest IC_50_ and exhibit cross reactivity against SARS-CoV-2 with A520S and H49Y spike mutations. This cross-reactivity is retained, even when mAbs are reverted to their germline sequences. This result suggests that some cross-reactivity may depend on properties of germline-encoded Abs. However, as the virus evolves, such “germline-encoded” Abs may become less effective. mAbs with high neutralization potency (mAbs 5, 12, 13, and 15) neutralized variants much less effectively than the original strain. Furthermore, reverting VH+VL sequences of those Abs to their germline configuration decreased neutralization potency against the reference strain but also against variant strains, suggesting that mutations helped with the establishment of cross-reactivity.

As a contrasting example, a virus that has accumulated higher levels of mutation, such as HIV, poses a far more challenging target to the immune system. Broadly neutralizing Abs against HIV are very infrequent in the primary repertoire and often harbor unusual sequence features (such as long CDR3s) and are typically highly mutated. Indeed, the canonical broadly neutralizing Abs against HIV, when reverted to their germline sequences, have significantly diminished neutralizing ability (37, 38). Our results indicate that, SARS-CoV-2 infection induces mutated Abs (mAbs 2, 5, 12, 13, and 15), albeit less so than broadly neutralizing Abs evoked by long-lasting HIV infection, that retain broadly neutralizing capacity, although at a reduced potency compared with the infecting strain neutralization potency. Very-high-affinity Abs that can broadly neutralize multiple virus variants are desirable as potential therapeutics because they would decrease the necessity of testing and producing mAb cocktails to neutralize many different virus variants.

Our results also reveal some properties of the virus-specific Ig clones that are highly relevant to immune memory. A subset of the spike-specific Ig clones encoded by IgG and IgA isotypes have properties we would expect to encounter in Ig genes of B cells engaged in active T-dependent Ab responses. Somatic mutations in IGHV genes of spike-specific IgG and IgA B cells increased with plasma neutralization titers. Thus, the frequency IgG or IgA clones with IGHV genes that exhibited 10% or higher levels of SHM increased with plasma neutralization titers. Furthermore, the neutralization potency of the 11 most powerful mAbs increased directly with the number of somatic mutations in the IGHV and IGLV genes. Our findings contrast with those of Yuan et al. (26) who compiled a list of 294 anti–SARS-CoV-2 RBD-targeting Abs identified by 12 different groups and found that 10% were encoded by IGHV 3-53 and had few somatic mutations. These authors also found that recognition of the ACE2-binding site was mediated by germline-encoded residues, suggesting that neutralization potency is dissociated from affinity maturation. It is possible that differences in the properties of Abs found by different groups and by us is due to the time of analysis relative to the beginning of disease and/or disease severity (39). It is also possible that the public SARS-CoV-2–reactive Abs that have been described to date are biased toward those that have low levels of SHM, because such Abs are easier to identify than mutated Abs with sequence variations. Furthermore, how generalizable our knowledge of public SARS-CoV-2 clones is to defining the overall immune response to SARS-CoV-2 is not yet clear, as we and others have shown that the vast majority of SARS-CoV-2–binding Abs are private (35, 40).

The Abs here reported were obtained from subjects 4–8 weeks from beginning of symptoms and who had disease severe enough to require hospitalization. Consistent with this view, Gaebler et al. (13) have recently found that Ab maturation persists during recovery. In their study, the number of mutations in VH and VL sequences increased in every 1 of the 6 individuals followed up to 6 months after their initial diagnosis even though their serum-neutralizing titers decreased over the same time interval, suggesting that the maturation of virus-specific Abs occurred in memory B cells. Studying the properties and ability of memory B cell responses to accommodate sequence variations in SARS-CoV-2 is important for our understanding of the durability of protective immunity in vaccinated and infected individuals.

## Methods

Further information can be found in Supplemental Methods.

### Experimental subjects.

Subjects were recruited from convalescent patients with COVID-19 followed at the University of Michigan Hospital or at the Henry Ford Health System. We have enrolled 12 convalescent patients recovering from COVID-19 infection. Eight patients were from Michigan Medicine and four were from the Henry Ford Health System. Subjects were between 29 and 73 years old and were free of virus (and symptoms) at the time of enrollment. Most had comorbidities, such as diabetes, obesity, and/or heart disease. Six had been seriously ill, requiring intensive care; five had been hospitalized but had not required intensive care; and one was not hospitalized and recovered at home. All blood samples were collected at least 4 weeks and less than 8 weeks after the beginning of symptoms, and patients were virus-free at blood collection. Heparinized blood samples were obtained, the plasma was collected and heat inactivated at 56°C for 1 hour. The original sample volume was restored by adding PBS and the PBMCs were isolated with Ficoll-Paque PLUS density gradient media (Cytiva, catalog 17-1440-02). Samples were frozen and stored in a nitrogen tank until posterior use.

### The 293T-ACE2 cell line.

To ensure optimal transduction, we produced a 293T cell line that overexpresses the SARS-CoV-2 receptor ACE2, 293T-ACE2 cells. Lentivirus packaging vectors psPAX2 (3.5 μg, Addgene, catalog 12260, RRID: Addgene_12260) and pC1-VSVG (3.5 μg, Addgene, catalog 1733, RRID: Addgene_1733) were cotransfected with 7 μg pLenti-ACE2 proviral plasmid using standard polyethyleneimine (PEI) precipitation methods. PEI precipitation was performed by incubating the plasmids with 42 μg PEI (molecular weight, 25,000 g/mole; Polysciences Inc., catalog 23966-1) in 1 mL Opti-MEM I Reduced Serum Medium (Gibco, catalog 31985070) at room temperature for 20 minutes, before adding it to 8 mL DMEM, high glucose media (Life Technologies, catalog 11965092) supplemented with 10% heat-inactivated FBS 1× Glutamax (Gibco, catalog 35050-061) and 100 U/mL Pen Strep (Gibco, catalog 15140-122). This DNA/PEI-containing media were then distributed to a T-75 flask containing 293T/17 cells (ATCC, CRL-11268, RRID: CVCL_1926). The lysate was collected after 72 hours and spun at 300*g* in a Beckman 5810R tabletop centrifuge for 10 minutes to pellet any cell debris. The supernatant containing Lenti-ACE2-VSV-G was then stored in aliquots at –80°C. A 6-well plate of 293T/17 cells was created 24 hours before transduction to attain 50% of confluence on the day it was transduced. The plate was then transduced with 1.5 mL per well of 1× Lenti-ACE2-VSV-G lentivirus supernatant on 5 wells, and the final well was maintained as a negative control, containing only DMEM 10% FBS 1× Glutamax, 100 U/mL Pen Strep. Twenty-four hours after transduction, the media were changed on all 6 wells, and after 48 hours of transduction, the media were supplemented with 3 μg/mL puromycin dihydrochloride (Gibco, catalog A1113803). The cells were cultured under selective pressure for 6 days, until the cells on the control well were completely dead. The selected cells were harvested and amplified under puromycin selection (1 μg/mL).

### Lenti-GF1 SARS-CoV-2 Wuhan Hu-1 and mutated spike-pseudotyped viruses.

Lentivirus packaging vectors psPAX2 (3.5 μg, Addgene, catalog 12260, RRID: Addgene_12260) and SARS-CoV-2–truncated spike envelope pSARS-CoV-2Δ19AA (35 μg) were cotransfected with 70 μg pGreenFire1(GF1)-CMV proviral plasmid (System Biosciences, catalog TR011VA-1) using standard PEI precipitation methods. The plasmid expresses both GFP and luciferase, allowing interchangeable detection of virus infected cells by fluorescence and luminescence readouts. PEI precipitation was performed by incubating the plasmids with 420 μg PEI in 10 mL Opti-MEM I Reduced Serum Medium at room temperature for 20 minutes, before adding it to 90 mL DMEM 10% FBS 1× Glutamax 100 U/mL Pen Strep. The DNA/PEI-containing media were then distributed equally to 5-T150 flasks containing 293T/17 cells. Supernatants were collected and pooled after 72 hours, filtered on a 0.45-micron GP Express filter flask (Millipore), pelleted by centrifugation at 26,400*g* on a Beckman Avanti J-E centrifuge at 4°C for 4 hours, and resuspended at 100× the original concentration (~1 × 10^7^ TU/ml) in DMEM. The lentivirus was stored in aliquots at –80°C. Alternatively, lentivirus Lenti-GF1-SARS-CoV-2Δ19AA truncated spike envelope displaying the H49Y, S247R, V367F, R408I, V483A, H519Q, A520S, and D614G mutations was produced using the same method. Variants were selected based on their prevalence in the wild and evidence of high infectivity or increased fitness and chosen to target the various domains of the spike protein, including the receptor-binding domain (H519Q, A520S).

### Neutralization assays.

293T/17, 293T-ACE2, and VeroE6 (VERO C1008, ATCC, catalog CRL-1586, RRID: CVCL_0574) cell lines were platted 24 hours before transduction to obtain 50% confluence on the day of transduction. To test efficiency of spike-pseudotyped virus transduction, concentrated virus or supernatant was added to cells in varying amounts. For neutralization assays 293T-ACE2 cells were used. Heat-inactivated plasma or mAb samples were serially diluted into DMEM 10% FBS, 1× Glutamax 100 U/mL Pen Strep. Plasma or Ab dilutions were then incubated with approximately 2.66 × 10^5^ TU/mL Lenti-GF1 virus pseudotyped with Wuhan Hu-1 SARS-CoV-2Δ19AA or mutant spikes for 30 minutes at room temperature before transduction. After neutralization, cells were transduced with the virus-plasma/Ab solution, virus and 10 μg/mL human IgG (Southern Biotech, catalog 0150-01), or virus alone and incubated at 37°C 5% CO_2_. Luminescence and GFP expression were analyzed 72 hours after transduction by luciferase assay, flow cytometry, and/or fluorescence microscopy. Assays were repeated at least 3 times, and IC_50_ was calculated from curves spanning 0% to 100% neutralization.

### Luciferase assay.

Chemiluminescence was detected using the Bright-Glo Luciferase Assay System (Promega, E2620) according to the manufacturer’s instructions. Briefly, the excess supernatant from the transduced plates was discarded, leaving 50 μL cell culture media; 50 μL Bright-Glo reagent equilibrated at room temperature was then added and the cells were incubated for 5 minutes to allow complete cell lysis. After incubation the cell lysates were transferred to 96-well white plates and read on a Synergy 2 plate reader (Biotek Instruments).

### Spike-specific B cell panning and single cell V(D)J analysis.

PBMCs from convalescent patients with COVID-19 were thawed. 1 × 10^6^ cells per mL of media were cultured in RPMI 1640 (Gibco, catalog 11875093) supplemented with L-Glutamine 10% FBS, 100 U/mL Pen Strep, 0.1% 2-mercaptoethanol, 5 μM CpG ODN 2006, 10 ng/mL CD40L, 50 ng/mL IL-2, 2 ng/mL, and IL-15 10 ng/mL IL-21 (cytokine cocktail media) (22) for 72 hours at 37°C 5% CO_2_. Cells were retrieved, resuspended at 1 × 10^6^ cells per mL in fresh cytokine cocktail media, and then transferred to wells coated with rabbit anti–Avi-tag Ab (4 μg/mL, GenScript, catalog A00674, RRID: AB_915553) bound to S1-C-6HIS-Avi (4 μg/mL, ABclonal, catalog RP01261) previously blocked with PBS 10% FBS. After a 24-hour incubation at 37°C 5% CO_2_, the plates were washed twice with RPMI 1640 supplemented with L-Glutamine 10% FBS, 100 U/mL Pen Strep 0.1% 2-mercaptoethanol at 37°C to remove nonbound cells. The cytokine cocktail media were then replenished, and the plates were cultured at 37°C 5% CO_2_ for an additional 48 hours. Bound cells were harvested by vigorous pipetting, and up to 10,000 cells were analyzed by Chromium Next Gel Bead-in-Emulsions (GEM) Single Cell V(D)J Technology. Briefly, cells were identified via generation of GEMs by combining barcoded Single Cell V(D)J 5′ Gel Beads v1.1, a master mix with cells (Chromium Next GEM Single Cell 5′ Library and Gel Bead Kit v1.1; 10× Genomics, catalog 1000165), and partitioning Oil on Chromium Next GEM Chip G (Chromium Next GEM Chip G Single Cell Kit; 10× Genomics, catalog 1000120). Reverse transcription and cDNA amplification were performed as recommended by the manufacturer. Next, the targeted enrichment from cDNA was conducted with the Chromium Single Cell V(D)J Enrichment Kit, Human B Cell (10× Genomics, catalog 1000016). The cDNA quality control analysis was carried out in an Agilent 2100 Bioanalyzer (Agilent Technologies) using the Agilent High Sensitivity DNA Kit (Agilent Technologies, catalog 5067-4626). The V(D)J enriched library was then constructed via Chromium Single Cell 5′ Library Construction Kit v1.1 (10× Genomics, catalog 1000166), and libraries were sequenced in a NovaSeq 6000 Sequencing System (Illumina). Chromium single-cell RNA-Seq output was processed in the Cell Ranger pipelines (10× Genomics, RRID:SCR_017344), and the V usage and clonotype profiles were generated and visualized by Loupe VDJ Browser.

### Bulk sequencing of IgH rearrangements from PBMCs.

IgH gene rearrangements were amplified from genomic DNA obtained from PBMCs and sequenced with an Illumina MiSeq, as previously described (24). For each subject, two biologically independent libraries each containing 100 ng input gDNA were generated and sequenced. For 2 of the subjects (1 and 101), spike-specific samples were additionally sequenced, each with 2 replicates.

### Analysis of bulk IGHV gene rearrangements from PBMC gDNA.

Sequences were paired and quality controlled with pRESTO (24), as described in ref. 41, and annotated with IgBLAST (42) using default parameters and the IMGT reference database (43). The resulting data were imported into ImmuneDB (44) for further downstream analyses.

### Ig heavy and light chain cloning and expression.

IgG heavy and light chain variable regions were cloned into human Igγ1, Igκ, and Igλ expression vectors (pFUSE2ss-CLIg-hK; Invivogen, catalog pfuse2ss-hclk) containing multiple cloning sites upstream of the human γ constant region (45). Eblock fragments for the variable regions were generated by IDT and had arms attached to allow for Gibson assembly. Eblock fragments were then inserted into their respective expression vectors using Gibson assembly (New England Biolabs) following the manufacturer’s instructions and transformed into NEB Stable Competent *E*. *coli* (New England Biolabs, catalog C3040I). Plasmid DNA was isolated from 2 mL cultures and mini prepped using a Qiagen QIAprep Spin Miniprep Kit (QIAGEN, catalog 27106). The sequence of insert was confirmed by Sanger sequencing from Eurofins using the following sequencing primer: pMT2-F 5′-TTGCCTTTCTCTCCACAGGT-3′. Heavy and light chain plasmids were cotransfected into 293T/17 cells using standard PEI precipitation methods. PEI precipitation was performed to obtain 9 mL mAbs, incubating 7 μg of both light and heavy chain plasmids with 42 μg PEI (molecular weight, 25,000 g/mole; Polysciences Inc., catalog 23966-1) in 1 mL Opti-MEM I Reduced Serum Medium at room temperature for 20 minutes, before adding it to 9 mL DMEM, high glucose 10% FBS 1× Glutamax 100 U/mL Pen Strep. The DNA/PEI-containing media were then distributed to a T-75 flask containing 293T/17 cells. The supernatant was collected after 72 hours and spun at 300*g* in a Beckman 5810R tabletop centrifuge for 10 minutes to pellet any cell debris before being transferred to a fresh tube. Alternatively, to obtain 60 mL mAb supernatant, 42 μg of light and heavy chain DNA were cotransfected after precipitation with 252 μg PEI in 1 mL Opti-MEM I Reduced Serum Medium, before adding to 60 mL DMEM, high glucose 10% FBS 1× Glutamax, 100 U/mL Pen Strep, distributed to 2 T-225 flasks containing 293T/17 cells.

### 50% tissue culture infectious dose assay.

For the 50% tissue culture infectious dose assay, 96-well plates were seeded with VeroE6 (VERO C1008, ATCC, catalog CRL-1586, RRID: CVCL_0574) at 20,000 cells per well for near confluence at 37°C for 24 hours. Plates and 460 μL Ab dilutions were transferred to BSL3 facility, and Ab solutions were incubated with 40 μL of SARS-CoV-2 USA-WA1/2020 clinical isolate viral solution for an MOI of 0.1. Ab. Virus solutions were incubated at 37°C for 1 hour. 100 μL of the viral solutions were diluted in 900 μL 2% FBS DMEM for –1 dilutions, and 10 μL of solution was added to 8 replicate wells of a 96-well plate for a –2 dilution. From the –2 dilution, serial dilutions were performed for a range of –2 to –7 for experimental conditions and –4 to –7 for untreated infection controls. Plates were incubated at 37°C for 3 days and then examined microscopically for visible cytopathic effect (CPE). Viral titer was then calculated from the number of CPE^+^ wells using a modified Reed and Muench method.

### Statistics.

All comparisons were done with GraphPad Prism 8 software (RRID:SCR_002798). Averages were compared by paired and unpaired 2-tailed *t* test, nonparametric Mann-Whitney test, or Wilcoxon’s test for paired analysis. A *P* value of equal to or less than 0.05 was considered significant. Nonlinear fit analysis of neutralization curves was used to determine IC_50_ and ID_50_ values using the GraphPad Prism “Absolute IC_50_, X is log (concentration)” option.

### Study approval.

Subjects were recruited from convalescent patients with COVID-19 followed at the University of Michigan Hospital or at the Henry Ford Health System. All subjects consented to these studies, and research was approved by the Institutional Review Boards at the University of Michigan and at the Henry Ford Health System.

## Author contributions

MC and JLP designed the research. MC, JLP, and MGDMB analyzed the results and wrote the manuscript. MGDMB, HL, DH, GS, BTE, ES, AAK, CM, TML, WM, and AMR performed experiments and helped in the analysis of results. TML, DG, AWT, CW, and ETLP planned or analyzed specific experiments, including those with live virus (AWT and CW), and sequencing and analysis of Ig sequences (ETLP). MS assisted with collection of patient samples and the evaluation of their clinical history. DG and ETK participated in the discussion and writing of the manuscript.

## Supplementary Material

Supplemental data

## Version 1 03/26/2021

In-Press Preview

## Version 2 05/10/2021

Electronic publication
