# Supplementary material for: IgV somatic mutation of human anti–SARS-CoV-2 monoclonal antibodies governs neutralization and breadth of reactivity
Source: JCI Insight. 2021 May 10;6(9):e147386. doi: 10.1172/jci.insight.147386 (PMC8262290; doi:10.1172/jci.insight.147386)
Supplement: Supplemental data [file jciinsight-6-147386-s268.pdf]

Supplementary Materials

Supplementary Methods

Spike pseudotyped virus titration

Lentivirus Lenti-GF1 pseudotyped with SARS-CoV-2 Δ19AA truncated spike envelope displaying the Wuhan-Hu-1, H49Y, S247R, V367F, R408I, V483A, H519Q, A520S and D614G mutations were tittered using fluorescence analysis. 293T-ACE2 cells were grown to 50% confluence on the transduction day (~200,000 cells). 100X concentrated stock of GF1 lentiviruses were then serially diluted from 10^{-1} to 10^{-6} and 300 µL of each dilution was added into each well. The plate was then incubated at 37°C 5% CO_2 atmosphere for 72 hours. After the incubation, the percentage of GFP-positive cells in each well was assessed by fluorescence microscopy. The virus titer was determined by counting GFP-positive cells in a total of 200 cells per field following transductions with serially diluted viruses according to the equation: Titer = (% of GFP-positive cells x number of transduced cells)/(dilution factor x total volume of the virus-media solution added to the well in mL). Representative images of the pseudotyped viruses-transduced and control cells were captured on an Olympus IX73 microscope with a GFP filter equipped with an X-cite series 120Q mercury light source with an Olympus DP21 computer.

Western Blotting

The expression of ACE2 in 293T/17, 293T-ACE2, and Vero E6 (VERO C1008 [Vero 76, clone E6, Vero E6; ATCC, Cat#CRL-1586, RRID: CVCL_0574) cell lines was assessed by western blotting. Briefly, cells were cultured, counted and lysed with RIPA Lysis Buffer System according to the manufacturer instructions (Santa Cruz Biotechnology, Cat#sc-24948A). Protein content was evaluated with Pierce BCA Protein Assay Kit (Thermo Scientific, Cat#23225) and 100 µg of protein extract was prepared with XT Sample Buffer, 4X (Bio-Rad, Cat#161-0791) and incubated at 95°C for 5 minutes. Samples ran 7.5% Mini-PROTEAN TGX Precast Protein Gels (Bio-Rad, Cat#4561024), were transferred to a PVDF membrane (Invitrolon PVDF Filter Paper Sandwich; Novex, Cat#LC2005) and the membrane was blocked with TBS 0.05% Tween 20 3% bovine serum albumin (BSA) 5% skim milk for 1 hour at room temperature. After

blocking, the membrane was incubated with mouse IgG2 anti-human ACE2 (5 μg/mL, OriGene, Cat#TA803841, RRID: AB_2627018) 2 hours at room temperature and mouse IgG1 anti-human GAPDH (0.2 μg/mL, Invitrogen, Cat#MA5-15738, RRID: AB_10977387) for 1 hour at room temperature. After washing, bands were detected by adding the alkaline phosphatase-conjugated antibody goat anti-mouse IgG (0.5 μg/mL; Southern Biotech, Cat#1030-04, RRID: AB_2794293) for 1 hour at room temperature. The reaction was visualized by subsequent addition of 5-bromo-4-chloro-3-indolylphosphate/nitro blue tetrazolium substrate (Sigma-Aldrich Cat#B5655-5TAB).

Flow Cytometry

Cells were washed with PBS without Ca^{2+} and Mg^{2+} trypsinized [Trypsin-EDTA (0.05%), phenol red; Gibco, Cat# 25300054], retrieved from the cell culture plates and transferred to 5 mL polystyrene round-bottom tubes (Falcon). Cells were washed 3 times with PBS 0.5% BSA 20 U/mL Sodium Heparin 0.1% Sodium Azide, 100,000 events were captured in a BD FACSCanto II (BD Biosciences, Franklin Lakes, NJ) and the number of GFP-positive cells and the GFP mean fluorescence intensity (MFI) were analyzed with FlowJo 10 software (FlowJo, LLC, Ashland, OR, RRID:SCR_008520).

Enzyme-Linked ImmunoSpot Assay for Detection of Immunoglobulin-Secreting Cells

Covid-19 convalescent patients PBMCs were thawed, 1 x 10^6 cells per mL of media were cultured for 5 days at 37ºC 5% CO_2, in RPMI 1640 (Gibco, Cat#11875093) supplemented with L-Glutamine 10% FBS 100 U/mL Pen Strep 0.1% 2-mercapthoethanol 5μM CpG ODN 2006 10 ng/mL CD40L 50 ng/mL IL-2, 2 ng/mL IL-15 10 ng/mL IL-21 (cytokine cocktail media) **(22)**, replacing the cytokine cocktail media on the third day. Ninety-six-well Filtration Plate MultiScreen HTS HA Sterile Plates (MilliPore, Cat#MSHAS4510) were activated and coated with 4 μg/mL goat anti-human IgG (Southern Biotech, Cat#2040-01, RRID: AB_2795640) for 1 hour at room temperature (IgG-secreting cells) or with 4 μg/mL rabbit anti-Avi-tag antibody (GenScript, Cat#A00674, RRID: AB_915553) for 1 hour at room temperature,

followed by 4 μg/mL S1-C-6HIS-Avi (ABclonal, Cat#RP01261) for 1 h room temperature (S1 spike subunit-specific IgG-secreting cells). After blocking overnight, cells were harvested, washed and resuspended in cytokine cocktail media, platted in serial dilutions and incubated at 37°C in 5% CO_2 atmosphere overnight. Spots of bound IgG were detected by adding the alkaline phosphatase-conjugated antibody goat anti-human IgG (0.5 μg/mL; Southern Biotech, Cat#2040-04, RRID: AB_2795643) for 1 hour at room temperature. The reaction was visualized by subsequent addition of 5-bromo-4-chloro-3-indolylphosphate/nitro blue tetrazolium substrate (Sigma-Aldrich Cat#B5655-5TAB). The number of spots was assed via a CTL ImmunoSpot S5 UV Analyzer equipped with ImmunoSpot ImmunoCapture and ImmunoSpot Counting softwares (Cellular Technology Ltd., Cleveland, OH, RRID:SCR_011082).

Enzyme-Linked Immunosorbent Assay for Detection of Human IgG

Nunc MaxiSorp ELISA plates (Thermo Fisher Scientific Cat# 44-2404-21) were coated overnight at 4°C with goat anti-human IgG heavy (4 μg/mL, Southern Biotech Cat#2040-01, RRID: AB_2795640) or goat anti-human IgG (H+L) (4 μg/mL, Southern Biotech, Cat#2015-01, RRID: AB_2795585). After blocking, the plates were incubated with serial dilutions of heat-inactivated human plasma or monoclonal antibodies. Bound IgG heavy, or kappa and lambda light chains were detected by adding goat anti-human IgG heavy-HRP (4 μg/mL, Southern Biotech, Cat#2040-05, RRID: AB_2795644), goat anti-human kappa-HRP (4 μg/mL, Southern Biotech, Cat#2060-05, RRID: AB_2795720) or goat anti-human lambda (4 μg/mL, Southern Biotech, Cat#2070-05, RRID: AB_2795753). The reactions were visualized by subsequent addition of 2,2′-Azino-bis (3-ethylbenzthiazoline-6-sulfonic acid) substrate (Southern Biotech, Cat#0202-01). All readings were recorded at 405 nm on a Synergy 2 plate reader (Biotek Instruments, Winooski, VT).

Supplementary Figures:

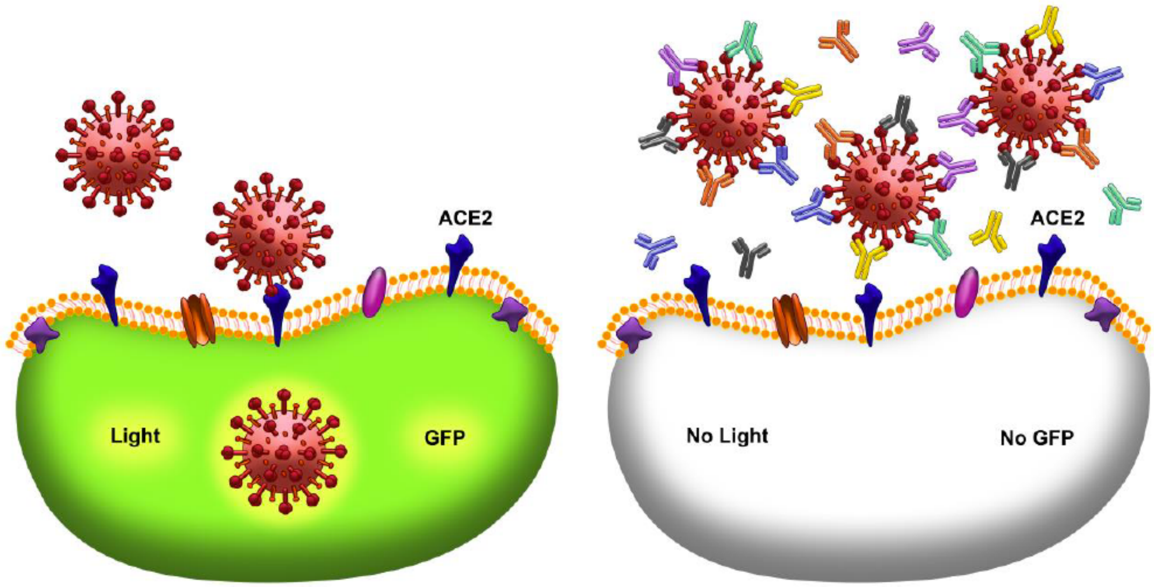

Figure S1. Schematic model of neutralization assay with SARS-CoV-2 spike pseudotyped virus. 293T cells expressing ACE2 receptor (293T-ACE2) were plated 24 hours prior to transduction in order to obtain 50% confluence. Plasma and monoclonal antibodies were diluted into cell culture media and incubated with Lenti-GF1virus pseudotyped with SARS-CoV-2Δ19AA spikes from Wuhan-Hu-1 or mutant strains, for 30 minutes at room temperature. After neutralization, cells were transduced with the virus-antibody solution or Lenti-GF1-VSV-G pseudotyped viruses and GFP expression and luminescence was analyzed 72 hours post transduction. In the absence of neutralizing antibodies, the pseudotyped viruses are able to infect the cells and induce the production of GFP and light via luciferase expression detected via fluorescent microscopy, flow cytometry and luciferase assay. Virus neutralization by the patients' plasma antibodies and monoclonal antibodies block the interaction of the Spike protein with ACE2 receptor on the cell surface, inhibiting infection and consequently GFP and luciferase expression.

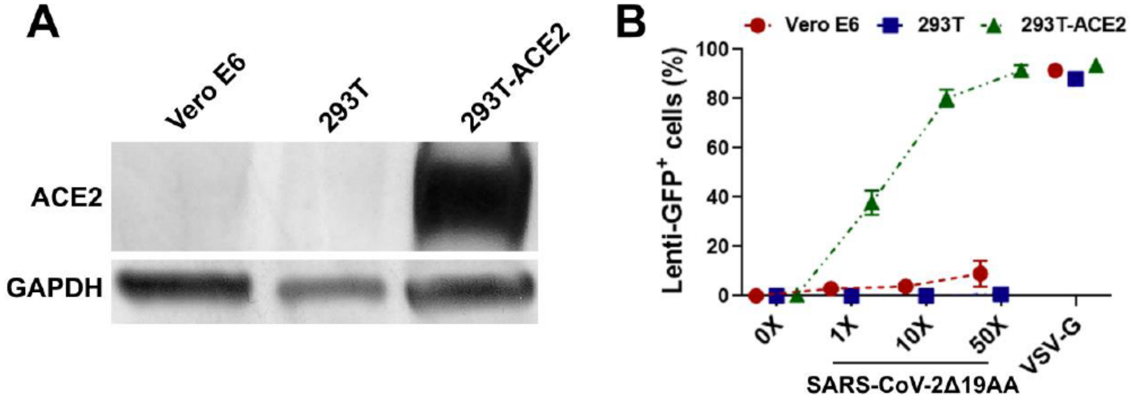

Figure S2. Efficiency of SARS-CoV-2 spike pseudotyped virus infection of various cell lines. (A) 293T cells were transduced with Lenti-ACE2-VSV-G and kept under selective pressure until the un-transduced control cells were completely dead. 293T-ACE2 selected cells were harvested, amplified and ACE2 and GAPDH expression was assessed via western blotting. **(B)** Vero E6, 293T, 293T cells expressing ACE2 receptor (293T-ACE2) were added to 24-well plates 24 hours prior to transduction in order to obtain 50% confluence. Cells were transduced with 50X, 10X or 1X GFP and luciferase-expressing SARS-CoV-2 spike or 1X VSV-G (Lenti-GF1-VSV-G) pseudotyped viruses. GFP expression was analyzed by flow cytometry 72 hours post-transduction.

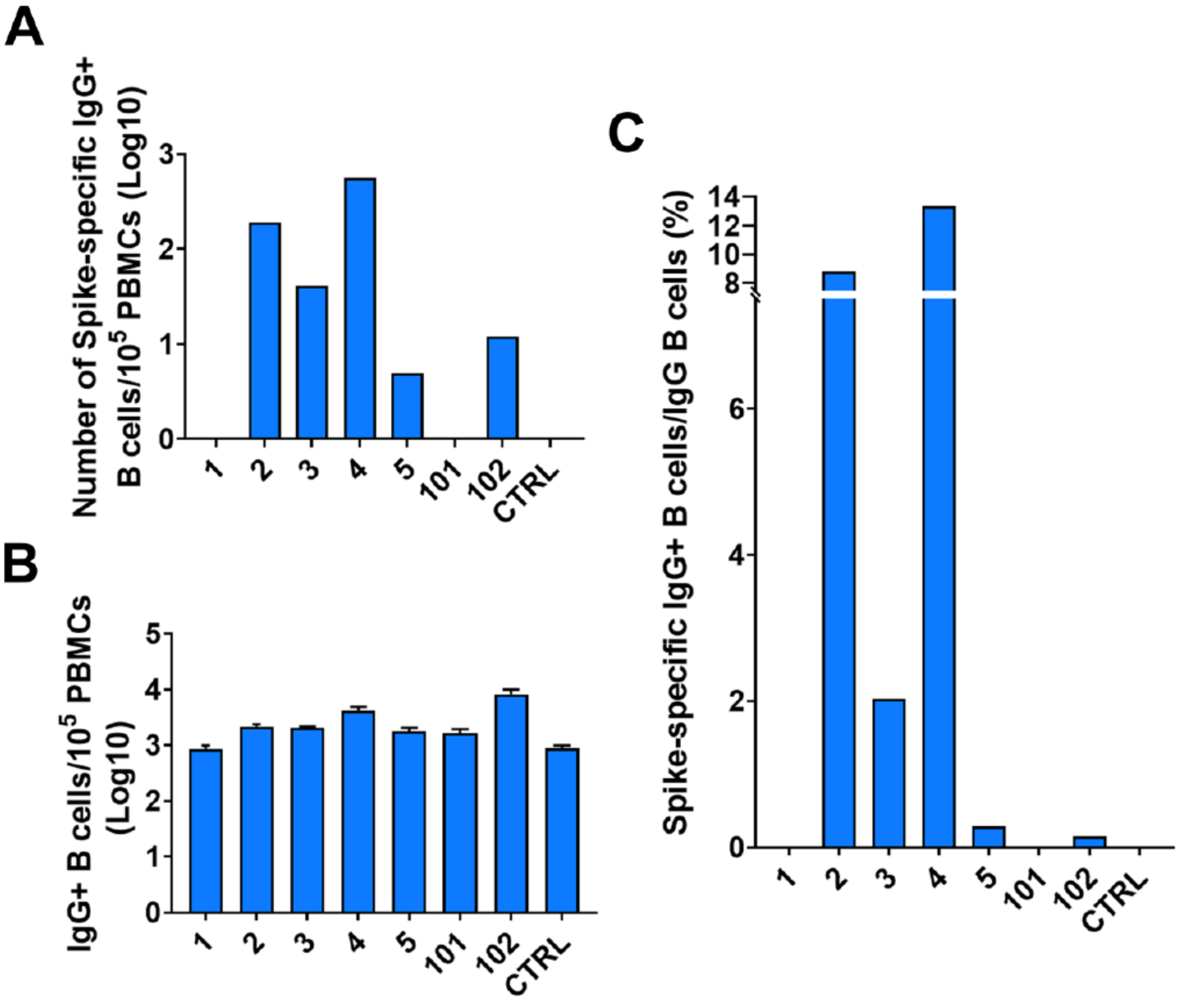

Figure S3. Frequency of spike-specific B cells in the peripheral blood of Covid-19 convalescent patients. **(A-C)** Covid-19 convalescent patients PBMCs were thawed and cultured for 72 hours in RPMI supplemented with 10% FBS, 100 U/mL Pen Strep ,0.1% 2-mercapthoethanol, 5µM CpG ODN 2006, 10 ng/mL CD40L, 50 ng/mL IL-2, 2 ng/mL, IL-15 and 10 ng/mL IL-21. Cells were then transferred to ELISPOT plates coated with **(A)** rabbit IgG anti-Avi-tag antibody and S1-C-6HIS-Avi or **(B)** goat anti-human IgG and incubated overnight at 37°C 5% CO_2 in the same media. IgG-producing cells were detected with goat anti-human IgG-AP and BCIP/NBT substrate. Graphs represent Mean±SEM of the number of **(A)** Spike-specific IgG-producing B cells, **(B)** IgG-producing B cells. **(C)** Graph represents the percentage of Spike-specific IgG-producing cells per total number of IgG-producing B cells in each patient.

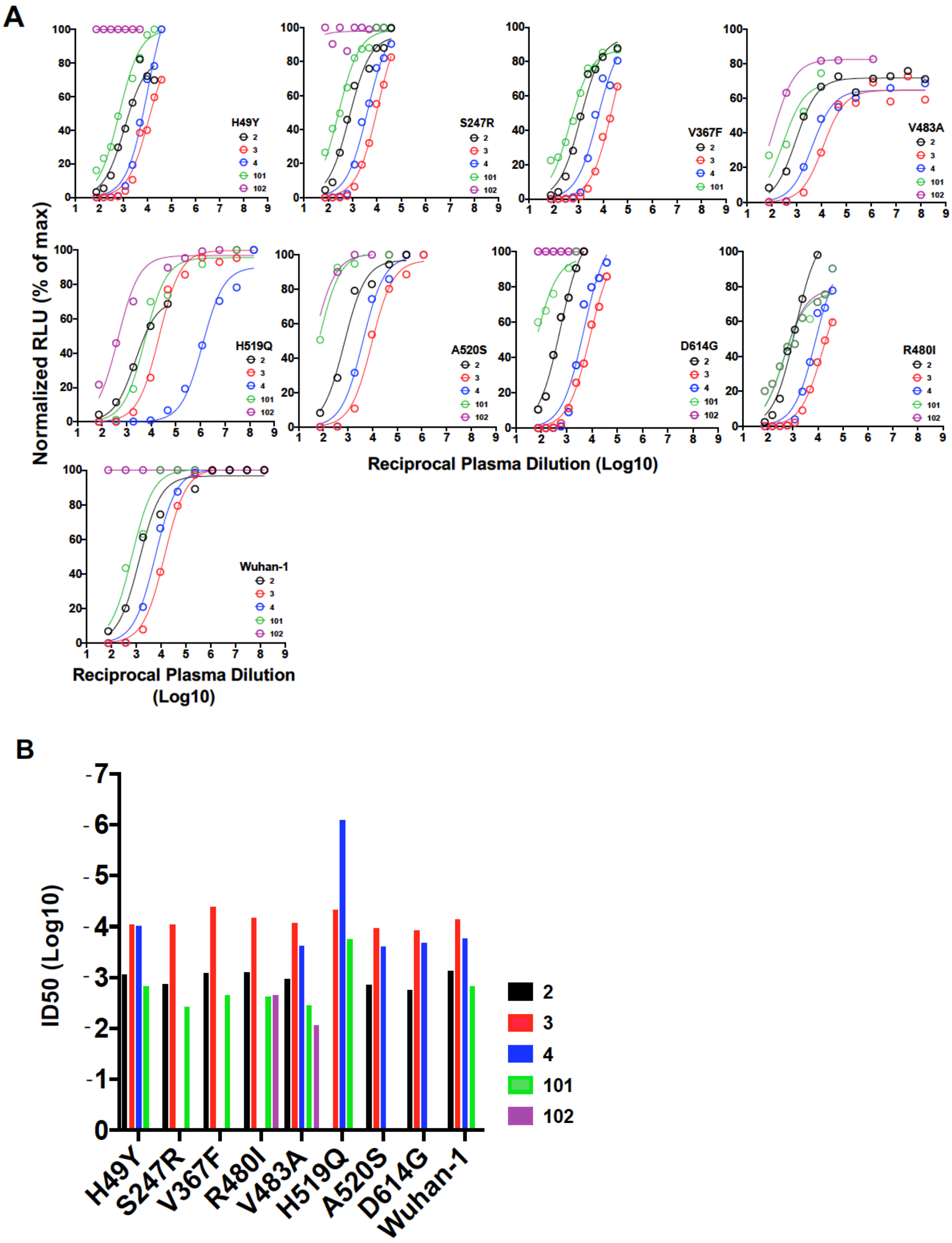

Figure S4. Neutralization of virus pseudo-typed with SARS-CoV-2 Wuhan-1 or mutant spikes by plasma of Covid-19 subjects. (A) Graphs depict virus neutralization curves obtained with serially diluted plasma from patients #2, #3, #4, #101 and #102 for SARS-CoV-2 Wuhan-1 spike or H49Y, S247R, V367F, R408I, V483A, H519Q, A520S and D614G mutant spikes. **(B)** Graph on the right represents the mean 50% inhibitory dilution (ID50) for Lenti-GF1 pseudo-typed with SARS-CoV-2 Wuhan-Hu-1 strain spike or H49Y, S247R, V367F, R408I, V483A, H519Q, A520S and D614G mutants.

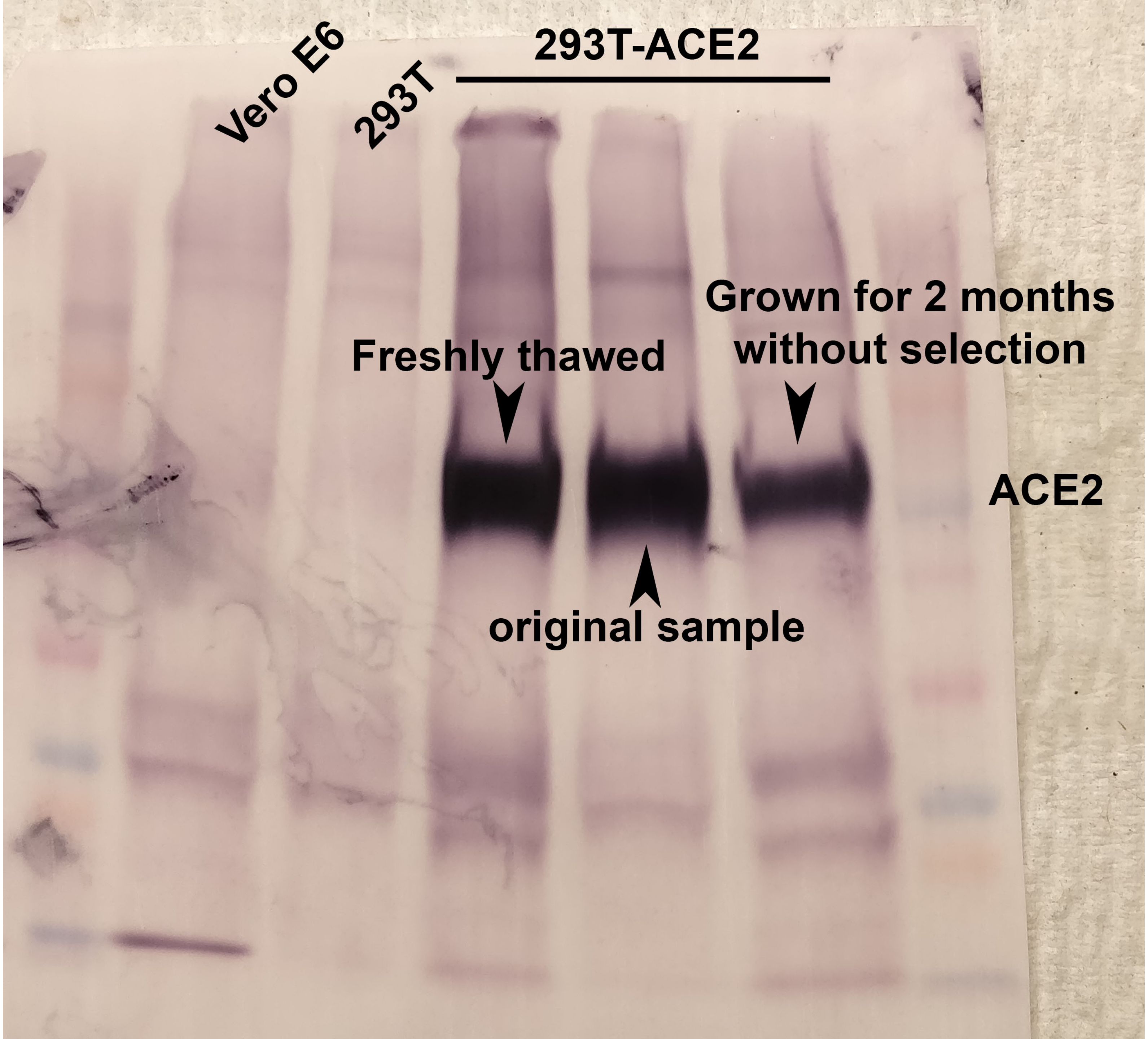
Vero E6
293T
293T-ACE2
Grown for 2 months
without selection
Freshly thawed
ACE2
original sample
